# Perceptions of an open visitation policy by intensive care unit workers

**DOI:** 10.1186/2110-5820-3-34

**Published:** 2013-10-17

**Authors:** Fernando José da Silva Ramos, Renata Rego Lins Fumis, Luciano Cesar Pontes Azevedo, Guilherme Schettino

**Affiliations:** 1 Research Laboratory of Anesthesiology and Intensive Care Medicine, Research and Education Institute of Sírio-Libanês Hospital, Cel Nicolau dos Santos 69, São Paulo 01308-060, Brazil

**Keywords:** Intensive care unit, Family, Visitation policy, Family centered care, Patient centered care

## Abstract

**Background:**

An intensive care unit (ICU) admission is a stressful event for the patient and the patient’s family. Several studies demonstrated symptoms of anxiety, depression, and posttraumatic stress disorder in family members of patients admitted to ICU. Some studies recognize that the open visitation policy (OVP) is related to a reduction in symptoms of anxiety and depression for the patient and an improvement in family satisfaction. However, some issues have been presented as barriers for the adoption of that strategy. This study was designed to evaluate perceptions of physicians, nurses, and respiratory therapists (RTs) of an OVP and to quantify visiting times in a Brazilian private intensive care unit (ICU).

**Methods:**

This observational and descriptive study was performed in the medical-surgical (22 beds) and neurologic ICU (8 beds) of Sírio-Libanês Hospital (HSL), São Paulo, Brazil. All physicians, nurses, and RTs from ICU were invited to participate in the study. A questionnaire was applied to all ICU workers who accepted to participate in the study. The questionnaire consisted of 22 questions about the visiting policy. During five consecutive days, we evaluated the time that the visitors stayed in the patient room, as well as the type of visitor.

**Results:**

A total of 106 ICU workers participated in this study (42 physicians, 39 nurses, and 25 RTs). Only three of the questions exposed a negative perception of the visiting policy: 53.3% of the participants do not think that the OVP consistently increases family satisfaction with patient’s care; 59.4% of ICU workers think that the OVP impairs the organization of the patient’s care; 72.7% of participants believe that their work suffers more interruptions because of the OVP. The median visiting time per day was 11.5 hours.

**Conclusions:**

According to physicians, nurses, and respiratory therapists, the greatest impact of OVP is the benefit to the patients rather than to the family or to the staff. Furthermore, they feel that they need communication training to better interact with family members who are present in the ICU 24 hours per day.

## Background

An intensive care unit (ICU) admission is a stressful event for the patient and the patient’s family. Several studies demonstrated symptoms of anxiety, depression, and posttraumatic stress disorder in family members of patients admitted to ICU [1-3]. In 1979, Molter published the Critical Care Family Needs Inventory [4]. Since then, other studies have focused on attendance to those needs [4-6]. An open visiting policy (OVP) in an ICU meets at least one of these needs: to be with the patient frequently. In the past several years, the search for improvement in patient care in a holistic way became evident. Patient-centered and family-centered care has been increasingly encouraged to improve the quality of care and the satisfaction of patients and their families. One of these proposals is to ensure an OVP to relatives of patients admitted to ICU [7,8].

In general, the time period of an ICU visit is described as restrictive or open/liberal. A restrictive policy allows family to visit during certain periods of the day and restricts the number of visitors per period. An OVP allows access to family at all times (24 hours), with or without restriction on the number of family members during any given period. An OVP is very common in the pediatric ICU setting but is still uncommon in an adult ICU [7,9-12].

Several studies developed in Europe and North America have demonstrated that most ICUs have a restrictive visiting policy [11-17]. Some studies recognize that the OVP is related to a reduction in symptoms of anxiety and depression for the patient and an improvement in family satisfaction [18]. Fumagalli et al. reported a reduction in cardiovascular complications with an OVP [19]. However, some issues have been presented as barriers for the adoption of that strategy [9,10,20-22]. An OVP could cause an increase in the workload for ICU workers and also create some delay in the performance of duties [10,22]. Nurses tend to be more skeptical about an OVP, despite recognition of the possible benefits to the patient [22].

In Brazil, there is no formal recommendation about the visiting policy in an ICU, and each institution is allowed to decide its own individual visitation strategy. Our institution adopted a 24-hour visitation policy 5 years ago (November 2008).

The objective of this study was to evaluate the perception of physicians, nurses, and respiratory therapists (RTs) regarding an OVP in one private ICU with 5 years of experience. Another objective of this study was to evaluate the length of stay of visitors in a patient’s room and the usual type of visitor.

## Methods

### Settings

This study was performed in the medical-surgical and neurologic ICU of Sírio-Libanês Hospital (HSL), a 380-bed tertiary-care hospital in São Paulo, Brazil. This ICU has 38 private rooms, which are divided into four different wings. Wings one and two are responsible for medical-surgical patients, wing three for cardiological patients, and wing four for neurological patients. A comfortable waiting room with several amenities is available for visitors. During the admission, the family receives an information leaflet that gives a general explanation about the ICU. In the past 5 years, we have adopted an OVP. Family members are told that they can visit the patient at any time during the day or night, and they also are allowed to sleep in the patient’s room (wing one in a bed and wings two through four in a rocking chair). During two periods, from 3 to 5 pm and 9 to 10 pm, up to two visitors are allowed in the patient room at the same time. During all other times, only one visitor is allowed in the room, but there are no restrictions about changing visitors. During invasive procedures (intubation, catheterization), family members are asked to stay out of the room. A large number of visitors are allowed if the patient is dying, especially during serious conflict cases. There is no regular hour for family conference. Only the patient’s representative can request information about the patient’s condition at any time, without restriction. Other relatives or visitors can ask for minor information at any time with the authorization of the patient’s representative.

The ICU has an open model of organization. A physician, who is not necessarily an intensive care specialist, is responsible for the patient, but there are many shared decisions with the ICU team about the patient’s care. The ICU team (wings one, two, and four) is made up of 38 intensive care physicians and 7 residents, 39 nurses, and 27 RTs. In addition, there are nurse assistants (NA) who are responsible for helping nurses with some patient care, such as bathing, eating, and drug administration. Intensive care physicians, nurses, and RTs are present in the ICU 24 hours per day, 7 days per week. During the day period, the shifts are 6 hours each, and during the night period, shifts are 12 hours each. The physician/patient ratio is 1:5 during the day and 1:10 at night; the nurse/patient ratio is 1:4 during the day and night; the RT/patient ratio is 1:5 during the day and night; the NA/patient ratio is 1:2 during the day and night. Family members or elderly assistants do not provide any part of the patient care.

### Study type

Observational and descriptive.

### Study design

All physicians, nurses, and RTs from the ICU (wings one, two, and four) were invited to participate in the study. ICU workers with less than 6 months employment in the institution were excluded. ICU workers from wing three were not included in this study because that wing belongs to another department. For all ICU workers who agreed to participate in the study, a questionnaire was filled out. The questionnaire (Additional file 1) contains data about demographic characteristics of ICU workers (age, gender, profession, length of experience in ICU, length of work in HSL), 20 questions relating to perceptions of an OVP, and two questions relating to communication training. Questions related to the impact of an OVP were written based on models used by Marco [21] and Garrouste-Orgeas [18]. All authors contributed to the adaptation of the questionnaire and tested the questions for this study. Questions related to ICU workers’ perceptions about an OVP presented four possible answers: never, occasionally, frequently, and always. The other three questions (numbers 20, 21, and 22) presented three possible answers: yes, no, or I don’t know. All ICU workers who agreed to participate in this study returned the questionnaire in a sealed envelope.

During five consecutive days, February 25 to March 1, 2013, we collected data about patients that were in the ICU or were admitted during in this period. For each patient, the following information was collected: age, gender, number of days in ICU before data collection, length of stay in ICU during data collection, SAPS3, and outcome. We also evaluated the time that the visitor stayed in the patient’s room, as well as the type of visitor (family or elderly assistance). This information was collected through the visits control that occurs in the ICU’s reception area. Each visitor is given a badge that lets us know the time of entry and time of exit from of the ICU. We did not verify the total number of visitors during the two time periods when it is allowed up to two visitors at the same time in the patient’s room.

This study was approved by the local ethics committee (nº HSL 2012/30).

### Statistical analysis

Collected data were analyzed by statistical software SPSS 13.0 (SPSS IBM, USA). Descriptive statistics for nominal data were expressed in proportions. Continuous variables normally distributed were described as mean and standard deviation. Median and interquartile range (IQR) was calculated for continuous variables that were not normally distributed. Each question has four possible answers that were scored from one to four (never, occasionally, frequently, and always). Because the questionnaire contained both positively and negatively formulated questions, we reversed the score given by the participants to the negative questions and computed the mean overall score for each question. Answers scored one and two were considered as a negative perception. For comparisons between physicians, nurses and RTs perceptions about a 24-hour visiting policy, the Kruskal-Wallis test was applied with Tukey HSD post-hoc analyses. For all statistical tests, a *p* value < 0.05 was considered statistically significant.

## Results

### Characteristics of physicians, nurses and respiratory therapists

Forty-two physicians participated in this study (35 assistants and 7 residents), one physician was excluded because time of work in the institution was less than 6 months, and two physicians were on vacations. All nurses participated in the study and two respiratory therapists were excluded because time of work in the institution was less than 6 months. Demographic characteristics of ICU workers are described in Table 1.

**Table 1:** Characteristics of the intensive care unit workers

**Variables**	**General (n = 106)**	**Physicians (n = 42)**	**Nurses (n = 39)**	**Respiratory therapists (n = 25)**
Age (yr), median (IQR)	33 (30–35.5)	34 (31–42)	31.5 (25–34)	33 (30.5–34.5)
Female gender, n (%)	69 (65,1%)	12 (28,5%)	35 (90%)	23 (92%)
ICU experience (yr), median (IQR)	6 (4–13)	6 (4–15)	6 (4–12)	9 (4–13)
Time of work in HSL ICU, (yr), median (IQR)	4 (2–6)	4 (2–6)	4 (1–5)	4 (3–9.5)

HSL, Sírio-Libanês Hospital; ICU, intensive care unit; IQR, interquartile range.

### Patients and visits

During the study period, 59 patients and their corresponding visitors were evaluated. Characteristics of patients, duration of visits, and type of visitors are described in Table 2. Median visiting time per day was 11.5 hours (IQR 6.3-17) and 79.7% of visitors were family members. Only two patients of this sample, whose stay in ICU was less than 2 days, did not have any kind of visit in the period of data collection.

**Table 2:** Characteristics of intensive care unit patients and type of visitors

**Variable**	**n = 59**
**Patient age (yr),** median (IQR)	73 (56–80)
**Female gender,** n (%)	25 (42.4%)
**SAPS3,** mean ± SD	39.2 ± 14.1
**Outcome (n%)**	
Discharge	53 (89.8)
Death	3 (5.1)
Inpatient	3 (5.1)
**Days of ICU before data collection**	3 (2–6)
Median (IQR)	
**LOS in ICU during data collection,**median (IQR)	2 (1–3)
**Visiting time, hours/day,**	11.5 (6.3-17)
median (IQR)	
**Type of visitor, n (%)**	
Family member	47 (79.7)
Elderly assistance	2 (3.4)
Family member + elderly assistance	8 (13.6)
None	2 (3.3)

SD, standard deviation; ICU, intensive care unit; IQR, interquartile range; SAPS3, Simplified Acute Physiology Score 3; LOS. length of stay.

### Perceptions about an OVP

**Figure 1:**
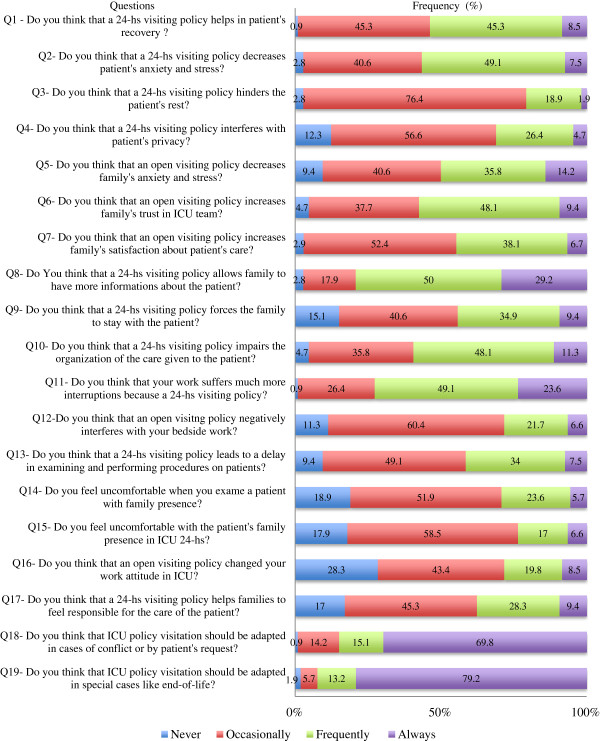
**Answers from ICU workers to the questionnaire about OVP.** (Questions 1 to 19). Q, question; ICU, intensive care unit; OVP, open visiting policy.

**Figure 2:**
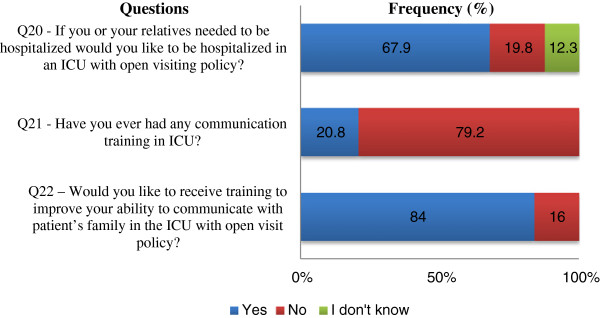
**Answers from ICU workers to the questionnaire about OVP.** (Questions 20 to 22). Q, question; ICU, intensive care unit; OVP, open visiting policy.

Figures 1 and 2 shows the questionnaire about OVP and the answers from all ICU workers who participated in the study. The answers to three of the questions exposed a negative perception of the visiting policy: 53.3% of the participants do not think that the OVP consistently increases family satisfaction with patient’s care; 59.4% of ICU workers think that the OVP impairs the organization of the patient’s care; 72.7% of participants believe that their work suffers more interruptions because of the OVP. Although 50% of the participants answered that an open visitation policy does not really decrease the family’s anxiety and stress, most of them (67.9%) would desire hospitalization in an ICU with an OVP if there ever was a necessity for that. When comparing different professionals, only two questions presented significant differences in perception: RTs indicated more frequently than physicians and nurses that an OVP hinders the patient’s rest; RTs also indicated more frequently that a 24-hour visiting policy leads to a delay in examining and performing procedures on patients, compared with nurses, but not compared with physicians (Table 3). In our ICU, only 20.8% of the workers have had some communication training, but 84% of the participants answered that they would like to receive communication training.

**Table 3:** Distribution of the ICU workers according to their answer to each question

**Question**	**Never**			**Occasionally**			**Frequently**			**Always**			
	**P%**	**N%**	**RT%**	**P%**	**N%**	**RT%**	**P%**	**N%**	**RT%**	**P%**	**N%**	**RT%**	***p *value**
1	0	2.6	0	47.6	41	48	45.2	48.7	40	7.1	7.7	12	0.96
2	0	2.6	8	35.7	41	48	57.1	46.2	40	7.1	10.3	4	0.2
3	4.8	2.6	0	81	87.2	52	14.3	5.1	48	0	5.1	0	0.001^#^&
4	14.3	10.3	12	59.5	53.8	56	21.4	30.8	28	4.8	5.1	4	0.75
5	9.5	7.7	12	38.1	35.9	52	40.5	38.5	24	11.9	17.9	12	0.33
6	2.4	0	16	38.1	43.6	28	54.8	41	48	4.8	15.4	8	0.70
7	2.4	0	8.3	59.5	51.3	41.7	35.7	41	37.5	2.4	7.7	12.5	0.46
8	2.4	0	8	16.7	15.4	24	54.8	51.3	40	26.2	33.3	28	0.44
9	9.5	20.5	4	40.5	46.2	32	33.3	25.6	52	16.7	7.7	0	0.22
10	2.4	7.7	4	40.5	41	20	50	35.9	64	7.1	15.4	12	0.09
11	0	0	14	26.2	30.8	20	57.1	41	48	16.7	28.2	28	0.48
12	4.8	15.4	16	64.3	64.1	48	28.6	12.8	24	2.4	7.7	12	0.4
13	9.5	12.8	4	50	56.4	36	35.7	23.1	48	4.8	7.7	12	0.03&
14	16.7	25.6	12	57.1	48.7	48	19	20.5	36	7.1	5.1	4	0.54
15	4.8	33.3	16	71.4	48.7	52	16.7	12.8	24	7.1	5.1	8	0.26
16	9.5	41	40	59.5	33	32	21.4	15.4	24	9.5	10.3	4	0.28
17	11.9	20.5	20	42.9	48.7	44	35.7	20.5	28	9.5	10.3	8	0.55
18	0	2.6	0	9.5	17.9	16	21.4	5.1	20	69	74.4	64	0.82
19	0	5.1	0	2.4	7.7	8	11.9	7.7	24	85.7	79.5	68	0.23
**Question**	**Yes**			**No**			**I don’t know**						
	P%	N%	RT%	P%	N%	RT%	P%	N%	RT%				
20	66.7	74.9	60	16.7	20.5	24	16.7	5.1	16				
21	40.5	7.7	8	59.5	92.3	92	-	-	-				
22	76.2	87.2	92	23.8	12.8	8	-	-	-				

Questions- correspondents numbers (see Figures 1 and 2 or Additional file 1). P, physician; N, nurse; RT, respiratory therapist. *p* < 0.05, Kruskal-Wallis test with Tukey HSD post-hoc analysis. ^#^Physicians vs. RT; &nurses vs. RT.

## Discussion

There is an increasing agreement in adoption of the philosophy that an open ICU policy is very important to both critically ill patients and family members.

This study has interesting findings. First, this is the first study that we know of which evaluated physicians’, nurses’, and RTs’ perceptions of an OVP in Brazil and Latin America. Second, the overall duration of visits was much longer than in previous reports of OVP. In our study, median visiting time was 11.5 hours per day. Fumagalli et al. reported a mean visiting time of 2.6 hours per day during an unrestricted visiting policy in a cardiology ICU [19]. Garrouste-Orgeas et al. analyzed visiting times from day 1 to day 5 after ICU admission, and the maximum median visit length was 120 minutes [18].

Our results suggest that there are major differences in the duration of ICU visits in different world regions. Not surprisingly, our families spend more time in an ICU, due to the fact that in Brazil families become very united in the face of serious disease and view as their responsibility to protect the patient from distress. Families play a major role in the decision-making process during the patient hospitalization.

Third, although the majority of ICU workers answered that an OVP policy impairs the organization of the care given to the patient and interferes with their work, they think that an open visitation policy helps the patient’s recovery by decreasing anxiety and stress. Regarding family members, the ICU staff did not report great benefit. For example, according to them the OVP does not always help to increase the family’s satisfaction or to decrease anxiety and stress. This partially could be explained by our model of work. Information may be given to a family representative at any time, but the patients’ doctors, who have full knowledge of the case, provide the most important information. With this model, the physicians on duty do not have a regular time for family conferences.

According to studies in other countries, the restricted visiting policies were preferred by the staff, especially by the nurses, because according to them, opening an ICU to visitors could interfere with their care process [22]. In our study, we found that the staff may feel uncomfortable when examining the patient with the family present and had complaints about the presence of the family for 24 hours per day. Some of the respondents appointed that OVP changed their work attitude in the ICU. Recent studies have demonstrated the effect of unrestricted visitation policies in ICUs and identified that OVP can make nurses and doctors feel controlled by the family’s presence or afraid to make an error and also may interfere with direct nursing / medical care [10,22]. Although our staff expressed positive statements about OVP, our results can help to identify the burdens and conflicts, which may assist other ICUs in determining solutions.

Despite the fact that there was a positive statement indicating that an OVP increases the family’s trust in the ICU team, they do not think that a longer family presence increases the satisfaction with the patient’s care. One reason for this feeling may be that even though the family has a perception that their relatives are receiving the best care, many more requests may be made by the family, which would create a burden of stress for the ICU team. This may sometimes lead to minor conflicts between ICU workers and families.

Fourth, we did not find substantial differences between physicians’, nurses’, and RTs’ perceptions about an OVP. In contrast with other studies, we did not find that nurses have a more skeptical opinion about the benefits of an OVP in ICU [10,22]. We also did not find that physicians have a more positive perception about it. One possible reason for those findings could be that the median time of work in HSL ICU is relatively low, 4 years (IQR 2–6), making an adaptation of this model of ICU visiting easier. As far as we know, this is the first study to describe and compare opinions of RTs. The RT group believed more frequently than the nurses and physicians that visitation hinders the patient’s rest and led to a delay in examining and performing procedures on patients. We did not find any other significant differences when comparing physicians and nurses. One possible reason for those differences is that RTs spend more time directly with the patient compared with nurses and physicians, as an RT session takes approximately 20–30 minutes per patient.

Another interesting finding is that, despite an existing OVP, in cases of end-of-life and serious conflicts, ICU workers were favorable of an adaptation in the visiting policy. Moreover, for all participants in this study there is a clear preference for self-hospitalization in an OVP.

The relationship between patient, ICU team, and family is extremely complex. This study demonstrates that our ICU team has a perception that an OVP is of benefit for the patient; however, this beneficial perception is not so clear with regards to the family. The perception of an increase in workload caused by the longer presence of the family in the ICU does not cause a negative perception of that visitation strategy.

Our study found that 79.2% of ICU staff members have gaps in communication training with families and 84% indicate a desire to have good family communication skills. The effectiveness of communication during daily rounds in the ICU can help staff organize their workload and improve their daily goals [23]. Lee et al. identified some strategies for implementation of improvement in the dynamics between staff and visitors [17]. According to this study, communication is one of three major themes that were identified and they encouraged formal communication skills training to facilitate implementation of an open visiting policy. We intend to introduce communication training for our team.

The present study has some limitations. First, the questionnaire was not formally validated, but it was built based on previous models. However, this does not invalidate our study, because the authors tested the questions, and during data collection there were no doubts reported about any questions. Furthermore, we did not make a qualitative interview with the ICU team. This approach could have helped to better understand the negative results. Second, this is a single-center study of a private Brazilian ICU and this model of OVP ICU with private rooms and organization is not representative of all Brazilian ICUs, although many ICUs are now changing to facilitate different visiting hours. In Brazil, the majority of ICUs are in public health facilities and are closed ICUs. Therefore, we cannot compare the perceptions of our ICU staff with others institutions that have very different characteristics. A third point is that a high level of severe burnout syndrome has been reported in ICU healthcare workers, and this was not explored by our study [24].

Finally, our research did not focus on the family and the patient’s perceptions. Future research efforts should be directed towards evaluating the impact that an OVP has on patient outcomes and family members.

## Conclusions

According to physicians, nurses, and respiratory therapists, the greatest impact of OVP is the benefit to the patient rather than to the family or the staff. Furthermore, the ICU staff feels that they need communication training to better interact with family members who are present in the ICU 24 hours per day.

## Abbreviations

RTs: respiratory therapist; OVP: open visitation policy; ICU: Intensive care medicine; HSL: Sírio-Libanês Hospital.

## Competing interests

The authors declare that they have no competing interests.

## Authors’ contributions

FJSR and RRLF designed the study, collected data, and prepared the manuscript; LCPA and GS designed the study and prepared the manuscript. All authors read and approved the final manuscript.

## Supplementary Material

Additional file 1 **Questionnaire.**1-22. Click here for file
