# Supplementary material for: Perceptions of an open visitation policy by intensive care unit workers
Source: Ann Intensive Care. 2013 Oct 17;3:34. doi: 10.1186/2110-5820-3-34 (PMC3854481; doi:10.1186/2110-5820-3-34)
Supplement: Additional file 1 — Questionnaire.1-22. [file 2110-5820-3-34-S1.docx]

Additional file 1. Questionnaire

Profession: ____________________ Gender: ______________ Age: __________________

Time of ICU experience: ________________________________________________________

Time of work in Sírio-Libanês Hospital: ____________________________________________

Q1 - Do you think that a 24-hs visiting policy helps in patient’s recovery?

( ) never ( ) occasionally ( ) frequently ( ) always

Q2- Do you think that a 24-hs visiting policy decreases patient's anxiety and stress?

( ) never ( ) occasionally ( ) frequently ( ) always

Q3- Do you think that a 24-hs visiting policy hinders the patient's rest?

( ) never ( ) occasionally ( ) frequently ( ) always

Q4- Do you think that a 24-hs visiting policy interferes with patient's privacy?

( ) never ( ) occasionally ( ) frequently ( ) always

Q5- Do you think that an open visiting policy decreases family's anxiety and stress?

( ) never ( ) occasionally ( ) frequently ( ) always

Q6- Do you think that an open visiting policy increases family's trust in ICU team?

( ) never ( ) occasionally ( ) frequently ( ) always

Q7- Do you think that an open visiting policy increases family's satisfaction about patient's care?

( ) never ( ) occasionally ( ) frequently ( ) always

Q8- Do You think that a 24-hs visiting policy allows the family to have more information about the patient?

( ) never ( ) occasionally ( ) frequently ( ) always

Q9- Do you think that a 24-hs visiting policy forces the family to stay with the patient?

( ) never ( ) occasionally ( ) frequently ( ) always

Q10- Do you think that a 24-hs visiting policy impairs the organisation of the care given to the patient?

( ) never ( ) occasionally ( ) frequently ( ) always

Q11- Do you think that your work suffers much more interruptions because a 24-hs visiting policy?

( ) never ( ) occasionally ( ) frequently ( ) always

Q12-Do you think that an open visiting policy negatively interferes with your bedside work?

( ) never ( ) occasionally ( ) frequently ( ) always

Q13- Do you think that a 24-hs visiting policy leads to a delay in examining and performing procedures on patients? ( ) never ( ) occasionally ( ) frequently ( ) always

Q14- Do you feel uncomfortable when you exame a patient with family presence?

( ) never ( ) occasionally ( ) frequently ( ) always

Q15- Do you feel uncomfortable with the patient's family presence in ICU 24-hs?

( ) never ( ) occasionally ( ) frequently ( ) always

Q16- Do you think that an open visiting policy changed your work attitude in ICU?

( ) never ( ) occasionally ( ) frequently ( ) always

Q17- Do you think that a 24-hs visiting policy helps families to feel responsible for the care of the patient?

( ) never ( ) occasionally ( ) frequently ( ) always

Q18- Do you think that ICU policy visitation should be adapted in cases of conflict or by patient's request?

( ) never ( ) occasionally ( ) frequently ( ) always

Q19- Do you think that ICU policy visitation should be adapted in special cases like end-of-life?

( ) never ( ) occasionally ( ) frequently ( ) always

Q20- If you or your relatives needed to be hospitalized would you like to be hospitalized in an ICU with open visiting policy?

( ) Yes ( ) No ( ) I don’t know

Q21- Have you ever had any communication training in ICU?

( ) Yes ( ) No

Q22- Would you like to receive training to improve your ability to communicate with patients’ family in ICU with open visiting policy?

( ) Yes ( ) No
